# Genomic and Metabolic Diversity of Marine Group I Thaumarchaeota in the Mesopelagic of Two Subtropical Gyres

**DOI:** 10.1371/journal.pone.0095380

**Published:** 2014-04-17

**Authors:** Brandon K. Swan, Mark D. Chaffin, Manuel Martinez-Garcia, Hilary G. Morrison, Erin K. Field, Nicole J. Poulton, E. Dashiell P. Masland, Christopher C. Harris, Alexander Sczyrba, Patrick S. G. Chain, Sergey Koren, Tanja Woyke, Ramunas Stepanauskas

**Affiliations:** 1 Single Cell Genomics Center, Bigelow Laboratory for Ocean Sciences, East Boothbay, Maine, United States of America; 2 Department of Biology, Colby College, Waterville, Maine, United States of America; 3 Department of Physiology, Genetics and Microbiology, University of Alicante, Alicante, Spain; 4 Josephine Bay Paul Center for Molecular Biology and Evolution, Marine Biological Laboratory, Massachusetts, United States of America; 5 Center for Biotechnology, Bielefeld University, Bielefeld, Germany; 6 Genome Science Group, Los Alamos National Laboratory, Los Alamos, New Mexico, United States of America; 7 Joint Genome Institute, Walnut Creek, California, United States of America; 8 Center for Bioinformatics and Computational Biology, University of Maryland, College Park, Maryland, United States of America; 9 National Biodefense Analysis and Countermeasures Center, Frederick, Maryland, United States of America; Max-Planck-Institute for Terrestrial Microbiology, Germany

## Abstract

Marine Group I (MGI) Thaumarchaeota are one of the most abundant and cosmopolitan chemoautotrophs within the global dark ocean. To date, no representatives of this archaeal group retrieved from the dark ocean have been successfully cultured. We used single cell genomics to investigate the genomic and metabolic diversity of thaumarchaea within the mesopelagic of the subtropical North Pacific and South Atlantic Ocean. Phylogenetic and metagenomic recruitment analysis revealed that MGI single amplified genomes (SAGs) are genetically and biogeographically distinct from existing thaumarchaea cultures obtained from surface waters. Confirming prior studies, we found genes encoding proteins for aerobic ammonia oxidation and the hydrolysis of urea, which may be used for energy production, as well as genes involved in 3-hydroxypropionate/4-hydroxybutyrate and oxidative tricarboxylic acid pathways. A large proportion of protein sequences identified in MGI SAGs were absent in the marine cultures *Cenarchaeum symbiosum* and *Nitrosopumilus maritimus*, thus expanding the predicted protein space for this archaeal group. Identifiable genes located on genomic islands with low metagenome recruitment capacity were enriched in cellular defense functions, likely in response to viral infections or grazing. We show that MGI Thaumarchaeota in the dark ocean may have more flexibility in potential energy sources and adaptations to biotic interactions than the existing, surface-ocean cultures.

## Introduction

Marine Group I (MGI) Thaumarchaeota [1]–[3] are numerically dominant and cosmopolitan within the ocean’s interior [4], [5]. Studies of the marine sponge symbiont *Cenarchaeum symbiosum* [6] and free-living, epipelagic *Nitrosopumilus maritimus* [7] have revealed that at least some members of this archaeal group fix inorganic carbon via a modified 3-hydroxypropionate/4-hydroxybutyrate (3H/4H) pathway [8], [9] and oxidize ammonia aerobically, and therefore may be responsible for a significant fraction of chemoautotrophic production in the dark ocean [10]. However, several studies have provided evidence for heterotrophy within the MGI [11], [12], suggesting a role for mixotrophy within this archaeal group. The potential importance of urea as a source of carbon and energy has also been demonstrated for deep water thaumarchaea of the Mediterranean Sea using PCR amplification [13] and in the Arctic Ocean through metagenomics [14]. In contrast, the cultured *N. maritimus* lacks the genes required for utilization of urea.

To date, representatives of MGI have not been successfully cultured from the dark ocean. To address this limitation, we employed a single cell DNA sequencing approach to obtain partial genomes of MGI from the mesopelagic region in the South Atlantic and North Pacific Oceans. Single amplified genomes (SAGs) representing MGI were found to be the dominant component of the mesopelagic archaeal community, with both ammonia monooxygenase (*amoA*) and nitrite reductase (*nirK*) genes successfully retrieved from a large percentage of the sequenced SAGs. The presence of urease genes within MGI SAGs confirms their potential for urea utilization in the mesopelagic, providing an alternative energy production pathway for these putative ammonia oxidizers. Genes supporting heterotrophic carbon assimilation were also identified, providing further evidence for mixotrophy among MGI in the dark ocean. While MGI SAGs share a large portion of genes with *N. maritimus* and *Cenarchaeum symbiosum*, a significant number of genes were present only in SAGs, greatly expanding the number of dark ocean thaumarchaeal protein families with genomic context.

## Materials and Methods

### Sample Collection and Construction of Single Amplified Genome (SAG) Libraries

No specific permission was required for the collection of water samples at HOT station ALOHA or the South Atlantic cruise station. None of the field collections involved endangered or protected species. GPS coordinates and water depths for all sample collections are provided in the manuscript.

Water samples for single cell analyses were collected from mesopelagic depths using Niskin bottles during cruises in the South Atlantic (27 November 2007, 800 m, Cruise KN192-5 station 11, 12°29′41.4″S, 4°59′55.2″W) and North Pacific (9 September 2009, 770 m; Hawaii Ocean Time-series (HOT) Cruise 215, station ALOHA; 22°45′N, 158°00′W) subtropical gyres. Replicate, 1 mL aliquots of water were cryopreserved with 6% glycine betaine (Sigma-Aldrich, St Louis, MO, USA) and stored at −80°C [15]. Single cell sorting, whole genome amplification, real-time PCR screens and PCR product sequence analyses were performed at the Bigelow Laboratory Single Cell Genomics Center (www.bigelow.org/scgc), as described previously [16]. PCR amplification of SSU rRNA and metabolic genes (*amoA*, *nirK*, *nifH*) from SAGs was done using primers and conditions provided in Table S1.

### Nitrite Reductase (nirK) Primer Design

Primer3 software [17] was used to design PCR primers for amplifying a portion of the nitrite reductase (*nirK*) gene from SAGs, using sequences retrieved from subphotic metagenomes and previously published *nirK* sequences (Figure S1).

### Phylogenetic Analysis of SAG SSU rRNA and Metabolic Genes

SSU rRNA and metabolic gene sequences were trimmed and edited using Sequencher v4.10.1 (Gene Codes, Ann Arbor, MI, USA). SAG SSU rRNA nucleotide and metabolic gene protein sequences were aligned with selected database sequences using MUSCLE v3.8 [18]. In order to reduce the number of misplaced gaps within metabolic gene sequence alignments, nucleotide sequences were translated to protein sequences and aligned, then backtranslated to produce nucleotide alignments using the RevTrans 1.4 server [19]. Maximum likelihood trees (1000 bootstrap replicates) for SSU rRNA and each metabolic gene nucleotide sequences were generated separately using RAxML version 7.0.3 [20] implemented within the ARB package [21]. MGI SSU rRNA SAG sequences with ≥99% similarity were grouped into phylotypes prior to tree construction (Table S2).

### SAG Sequencing and Analysis

A total of 37 MGI Thaumarchaeota SAGs were chosen for whole genome sequencing based on multiple displacement amplification (MDA) kinetics, presence of metabolic genes from PCR screening and geographic location. Three approaches were used for sequencing MGI SAGs (Table S3): 1) A combination of Illumina and 454 shotgun sequencing (AAA007O23), or Illumina only (AAA001A19), as described in Swan et al. [16], 2) a combination of Illumina and PacBio long read sequence data (AAA007N19, AAA288I14, and AAA288J14) as described in Martinez-Garcia et al. [22] and assembled using Velvet-SC [23] and PBcR [24], and 3) 454 shotgun sequencing of Nextera-prepared libraries followed by dual assembly with Newbler v2.4 and Geneious Pro v.5.5.6 [25] (all remaining SAGs; total of 32). For each of 32 Single Amplified Genomes (SAGs), raw 454 sequences were trimmed in Geneious Pro v5.5.6 and any remaining Nextera transposon insert sequences were removed using TagCleaner v0.11 [26]. Sequences were then assembled separately in Newbler v.2.4 (Roche) using default settings and Geneious using the high-sensitivity setting. The Newbler-assembled were imported into Geneious and co-assembled with both the Geneious-assembled contigs and the unused reads. The dual assembled contigs and all other contigs longer than 300 bp were pooled and annotated. Nextera-prepared sequencing libraries were generated using the Roche Titanium-Compatible kit and MDA product as the input DNA, following the manufacturer’s instructions [27]. A total of 32 Nextera sequencing libraries constructed from SAGs were barcoded and sequenced (454 FLX Titanium chemistry) on 1/2 microtiter plate. A metagenome library from the South Atlantic sampling station at 800 m was also prepared using the Nextera kit and DNA extracted from a collected water sample (Table S4). Whole-genome sequence data for MGI SAGs are available in IMG under accession numbers listed in Table S3.

To estimate the completeness of each assembled SAG genome, we analyzed all finished genome sequences within the archaeal domain (n = 155) available from the IMG [28]. Based on COG gene classifications, a set of conserved single copy genes (CSCGs) were extracted from these finished archaeal genomes. A CSCG was defined as a gene that occurs only once in each of 98% of the genomes that contributed to the taxonomic group. The number of archaeal CSCGs was 94. The ratio of the number of CSCGs observed for each SAG assembly, and the finished archaeal genomes, was used as a measure of genome recovery (Table S3).

The gene modeling program Prodigal (http://prodigal.ornl.gov/) was run on the draft single cell genomes, using default settings that permit overlapping genes and using ATG, GTG, and TTG as potential starts. The resulting protein translations were compared to the GenBank non-redundant database (NR), the Swiss-Prot/TrEMBL, Pfam, TIGRFam, Interpro, KEGG, and COGs databases using BLASTP or HMMER. From these results, product assignments were made. Initial criteria for automated functional assignment set priority based on TIGRFam, Pfam, COG, Interpro profiles, pairwise BLAST versus Swiss-Prot/TrEMBL, and KO groups. The annotation was imported into the Joint Genome Institute Integrated Microbial Genomes (IMG; http://img.jgi.doe.gov/cgi-bin/pub/main.cgi) [29].

### Metagenome Recruitment Analysis

The basic approach of Rusch et al. [30] was used to estimate abundances of metagenome sequences that are close relatives of MGI SAGs and marine thaumarchaea cultures within each metagenome depth profile (Table S4). BLASTN+ v2.2.25 was used to recruit metagenome sequences to each SAG assembly using default parameter values, except for the following: -evalue 0.0001 -reward 1 -penalty -1 -soft_masking true -lcase_masking -xdrop_gap 150. The percentage of unique recruits (≥200 bp in length and matching at ≥95% identity) from each metagenome matching to each SAG was normalized by genome assembly length. Metagenomes used in the recruitment analysis were gathered from previously published studies and a metagenome sequenced in this study (SA, South Atlantic Gyre; Table S4). Genome abundances determined for each metagenome were calculated from BLAST output and plotted using custom R scripts. Metagenomes used in fragment recruitment analysis were quality processed using PRINSEQ [31] and all sequences identified with the following characteristics were removed from further analysis: sequences <100 bp, sequences containing any ambiguities (N’s), all forms of replicate and duplicate sequences, and sequences with a minimum entropy value of 70 (applied to pyrosequencing datasets only).

### Protein Homology and Identification of Genomic Islands

A non-redundant set of protein sequences from all 37 MGI SAGs was used as input for the calculation and visualization of sequence homology between *C. symbiosum* and *N. maritimus* protein sequences by BLAST Score Ratio analysis [32]. Genomic islands within this non-redundant protein set were determined by first using BLASTN-based recruitment as described above, using a 454 metagenome set consisting of sequences from HOT station ALOHA (500 and 770 m) and the subtropical South Atlantic (800 m) (Table S4). Protein sequences with zero recruitment across the entire sequence length were identified as putative genomic islands. This conservative measure was employed to avoid the inclusion of false positives.

### Nucleotide, Genome and Metagenome Accession Numbers

MGI SAG SSU rRNA and metabolic gene sequences have been deposited in GenBank with the following accession numbers: SSU rRNA, HQ675727–HQ675855; *amoA*, JF719126–JF719207; *nirK*, JF719208–JF719271. Whole-genome sequence data for 37 MGI Thaumarchaeota SAGs used for our analyses are available in IMG under accession numbers listed in Table S3. Metagenome sequence data for the subtropical South Atlantic are available under the MG-RAST ID 4547679.3 (“South_Atlantic_Gyre_Metagenome).

## Results and Discussion

### Community Composition, Metabolic Gene Diversity and Depth Distribution of Archaeal SAGs

**Figure 1:**
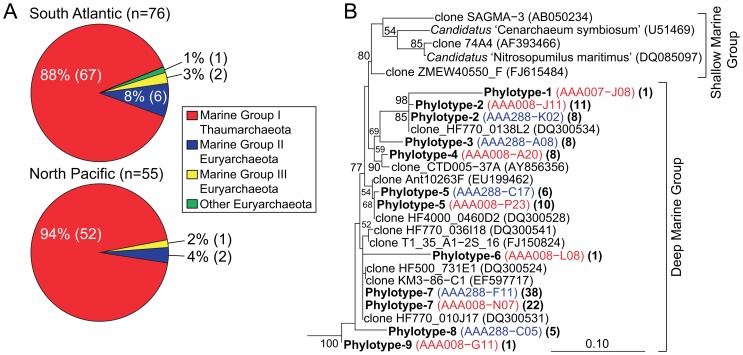
Phylogenetic analysis of archaeal single amplified genomes (SAGs) from South Atlantic and North Pacific gyres. The phylogenetic composition of archaeal SAG libraries (**A**) and an inferred phylogenetic tree of partial SSU rRNA sequences (Table S2) amplified from Marine Group I (MGI) Thaumarchaeota SAGs (**B**). Numbers in parentheses represent the number of SAGs in each archaeal group. The tree was inferred using maximum likelihood in RAxML and bootstrap (1000 replicates) values ≥50% are indicated at nodes. Sequences from South Atlantic SAGs are colored red, and North Pacific (HOT station ALOHA) SAG sequences colored blue. MGI Thaumarchaeota sequences with ≥99% similarity were grouped into phylotypes (bold), representative sequence(s) are in colored parentheses, and total number of sequences in each phylotypes is in parentheses (bold).

A total of 1,252 and 630 SAGs from the South Atlantic and North Pacific Ocean, respectively, were screened for archaeal SSU rRNA genes and MGI was found to be the dominant archaeal group in both ocean regions (Figure 1A). Several SAGs affiliated with Euryarchaeota Marine Group II [33] and Group III [1] clusters were recovered from each site. Phylogenetic analysis of MGI SAG and reference SSU rRNA gene sequences revealed shallow marine samples and cultures were more distantly related to those from the dark ocean (Figure 1B). A total of nine phylotypes (≥99% SSU rRNA similarity) were defined for MGI SAGs, and six phylotypes comprised of more than one sequence included SAGs from both ocean regions (Table S2). These results support previous studies showing that the dark ocean archaeal community is composed mostly of MGI [34], [35]. The high degree of similarity between SSU rRNA sequences recovered from SAGs collected from two geographically distinct regions highlights the cosmopolitan nature of this group, although significant genomic differences between cells may exist that are not evident from SSU rRNA similarity [36].

Gene sequences of *amoA* were successfully PCR-amplified from over 50% of MGI SAGs from which the 16S rRNA gene was also recovered (Table 1). Phylogenetic analysis of *amoA* sequences revealed a consistent clustering of SAG *amoA* sequences with environmental sequences belonging to the ‘Water Column B Group’ cluster that have been retrieved from samples within dark ocean regions (Figure S2; [6], [35], [37], [38]). These results support the potential for ammonia oxidation among the majority of MGI SAGs recovered from the mesopelagic.

**Table 1:** Metabolic genes amplified from Marine Group I (MGI) Thaumarchaeota single amplified genomes (SAGs).

			Marine Group I (MGI) PCR screening^1^	
Station	Depth (m)	No. of SAGs	*amoA*	*nirK*
South Atlantic	800	67	40 (60%)	32 (48%)
North Pacific	770	52	42 (81%)	32 (62%)
Total		119	82 (69%)	64 (54%)

1 Total number and percentage of SAGs containing listed genes: *amoA*, ammonia monooxygenase subunit A; *nirK*, nitrite reductase.

PCR screening also recovered *nirK* from the majority of MGI SAGs (Table 1), suggesting this gene plays an important metabolic role in the putative ammonia oxidizing archaea (Figure S2). MGI SAG *nirK* sequences formed a monophyletic group consisting exclusively of sequences recovered from marine habitats, and were most similar to a bathypelagic fosmid sequence recovered from station ALOHA (Figure S3). Conserved sites corresponding to copper coordinating residues were present in SAG *nirK* sequences, distinguishing them from multicopper oxidases (MCOs) identified in genomes of *C. symbiosum* and *N. maritimus* (Figure S1). Denitrifying bacteria utilize the *nirK* gene for the conversion of nitrite (NO_2_^−^) to nitric oxide (NO); however, this gene has also been identified in aerobic, autotrophic ammonia-oxidizing bacteria and archaea [6], [7], [39]–[42]. Although the exact role of denitrification genes in nitrifiers is still unknown, the presence of *nirK* and its associated activity has been shown to support growth in some ammonia-oxidizing bacteria [43], [44], as well as provide a mechanism for NO_2_^−^ tolerance [45]. Recently, production of nitrous oxide (N_2_O) by ammonia oxidizing archaea in the surface ocean has been shown to be a globally important process [46], and our results suggest that N_2_O produced via ‘nitrifier-denitrification’ may also occur in the dark ocean.

**Figure 2:**
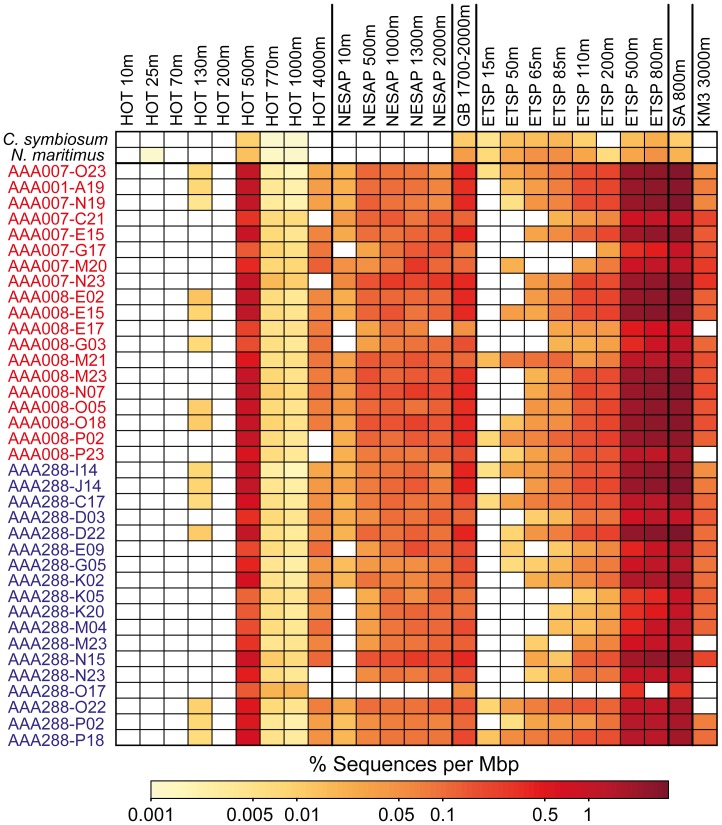
Depth distribution of single amplified genome (SAG)-related thaumarchaea determined by metagenomic fragment recruitment. Thaumarchaea cultures and SAGs are listed along the y-axis and metagenomes are listed along the x-axis. SAGs are colored according to source; red, South Pacific; blue, North Pacific. The scale bar indicates the percentage of aligned metagenome sequences that had ≥95% nucleotide sequence identity and an alignment length ≥200 base pairs for the BLASTN-based recruitment, normalized by the length of each genome. *C. symbiosum*, *Cenarchaeum symbiosum*; *N. maritimus*, *Nitrosopumilus maritimus*; HOT, Hawaii Ocean Time Series station ALOHA; NESAP, North Eastern Subarctic Pacific; GB, Guaymas Basin hydrothermal vent plume; ETSP, Eastern Tropical South Pacific; SA, Subtropical South Atlantic; KM3, Ionian Sea Station KM3.

The abundance of MGI gene sequences in several metagenome depth profiles, determined by fragment recruitment analysis, revealed a strong depth-dependent distribution for dark ocean MGI SAG relatives (Figure 2 and Table S4). MGI SAG recruitment was highest in waters below the photic zone (∼200 m) at all geographic regions. In contrast, recruitment of sequences from cultures of *C. symbiosum* and *N. maritimus* was lower than MGI SAGs at all depths and exhibited some depth-dependence only at HOT station ALOHA in the North Pacific Ocean (Figure 2). The highest recruitment of MGI SAGs was found at the 500 m depth at station ALOHA and depths below 200 m at the eastern tropical South Pacific (ETSP) station. Whereas the 500 m depth at ALOHA is located above the oxygen minimum zone, ETSP stations deeper than 50–100 m are below the oxycline, and the 500 m station is within the core of the oxygen minimum zone (OMZ) [47]. Transcript sequences matching to genes from *N. maritimus* were dominant among the RNA fraction retrieved in ETSP upper waters (15–85 m) [47]. Although the fragment recruitment analysis employed here is based on genomic DNA, which does not account for gene transcription activity, our results indicate that MGI may also be active within the OMZ core. The depth distributions of MGI SAGs further highlight the importance of nitrification and associated nitrogen-based metabolic processes occurring within both aerobic and low-oxygen dark ocean regions [48].

### Genomic Characteristics and Metabolic Potential of MGI Thaumarchaeota SAGs

Genome recovery estimates of MGI SAGs ranged from 2.1 to 94.7%, with genome size estimates of 0.72 to 2.73 Mbp (Table S3). Thus, MGI SAG genome sizes are similar to those found for *N. maritimus* (1.65 Mbp) and *C. symbiosum* (2.05 Mbp). GC content of MGI SAGs (33.56–36.46%) was also comparable to *N. maritimus* (34.20%), but significantly lower than *C. symbiosum* (57.70%).

**Figure 3:**
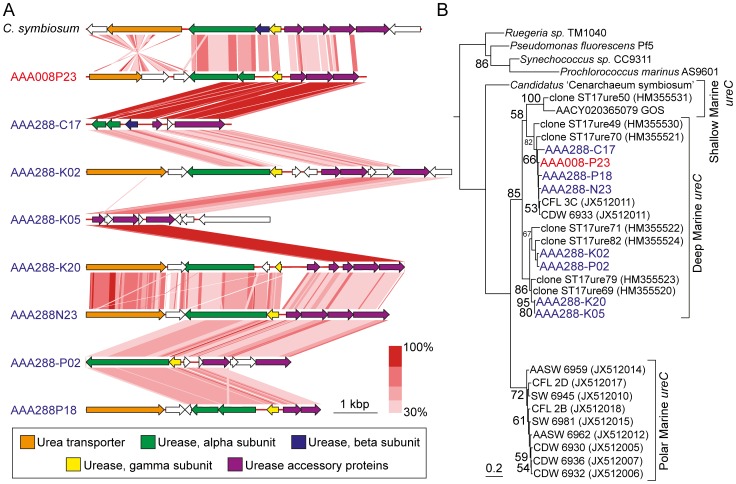
Syntenic and phylogenetic analysis of genes involved in urea hydrolysis. Arrangement and similarity of genes involved in the urea hydrolysis pathway *C. symbiosum* and SAGs (**A**), and an inferred phylogenetic tree of α-subunit of urease (*ureC*) gene sequences from SAGs and selected cultures and environmental samples (**B**). SAGs are colored according to source; red, South Pacific; blue, North Pacific. The scale bar indicates tblastx similarity values between genes. The tree was inferred using maximum likelihood in RAxML and bootstrap (1000 replicates) values ≥50% are indicated at nodes. *C. symbiosum*, *Cenarchaeum symbiosum*.

Genes encoding all three subunits of ammonia monooxygenase (*amoABC*) were identified within the majority of MGI SAGs with highest genome completeness (Table S5). In addition to nitrite reductase (*nirK*), a number of copper binding proteins within the plastocyanin/azurin family and multicopper oxidases (MCOs) were identified. Plastocyanin/azurin blue type (I) copper proteins within *N. maritimus* have been implicated as electron carriers for an alternative mode of ammonia oxidation than that found in bacteria [7]. Although the exact details of ammonia oxidation employed by thaumarchaea remain unclear, genes identified within MGI SAGs suggest that dark ocean thaumarchaea utilize a similar strategy identified in *N. maritimus* for carrying out the first step in nitrification for energy production. Genes involved in the transport and hydrolysis of urea were also identified in several MGI SAGs and found to be syntenic to genes identified in *C. symbiosum* (Figure 3A). While PCR-amplified sequences of the urease α-subunit (*ureC*), as well as several subunits from fosmid sequences have previously been retrieved from the dark ocean [13], [49], this is the first identification of urease genes within putatively free-living dark ocean MGI assembled genomes. Phylogenetic analysis of *ureC* sequences from MGI SAGs revealed they are associated with a subclade consisting of other deep ocean sequences (Figure 3B). The role of urea in supporting thaumarchaeal production in polar waters was recently reported [14], and our results suggest this metabolic pathway may have global importance for thaumarchaea within the dark ocean.

A majority of gene homologs involved in the modified 3H/4H pathway [50] were identified in MGI SAGs with larger genome assembly sizes (Table S6). Five genes within the described 3H/4H pathway had no homology to gene sequences from MGI SAGs and the *N. maritimus* genome; however, several proteins (e.g., alcohol dehydrogenases, aldehyde dehydrogenases, acyl-CoA synthetases) identified in *N. maritimus* were proposed to provide similar function and were also identified in MGI SAGs. In addition to autotrophic growth, enzymes supporting a near-complete oxidative tricarboxylic acid (TCA) cycle were also identified, suggesting a role for this pathway in biosynthesis (Table S6). Similar to *C. symbiosum*, genes supporting the Embden–Meyerhof–Parnas (EMP) and nonoxidative pentose phosphate pathways were identified, supporting gluconeogenesis and sugar production for biosynthesis.

Several ABC transporter classes were identified in MGI SAGs involved in the transport of metals, sugars, phosphonate, and antibiotics (Table S7). Although the presence of phosphonate transporters could suggest a role for these organic compounds as a source of phosphorus, genes for the production of C-P lyases and hydrolases were not identified in MGI SAGs. Genes supporting motility were also not identified in MGI SAGs, and although incomplete genome recovery is problematic for the interpretation of missing genes, it is statistically unlikely genome incompleteness can explain the absence of these genes from all genomes [51].

### Protein Family Expansion and Characterization of Genomic Islands within MGI Thaumarchaeota SAGs

**Figure 4:**
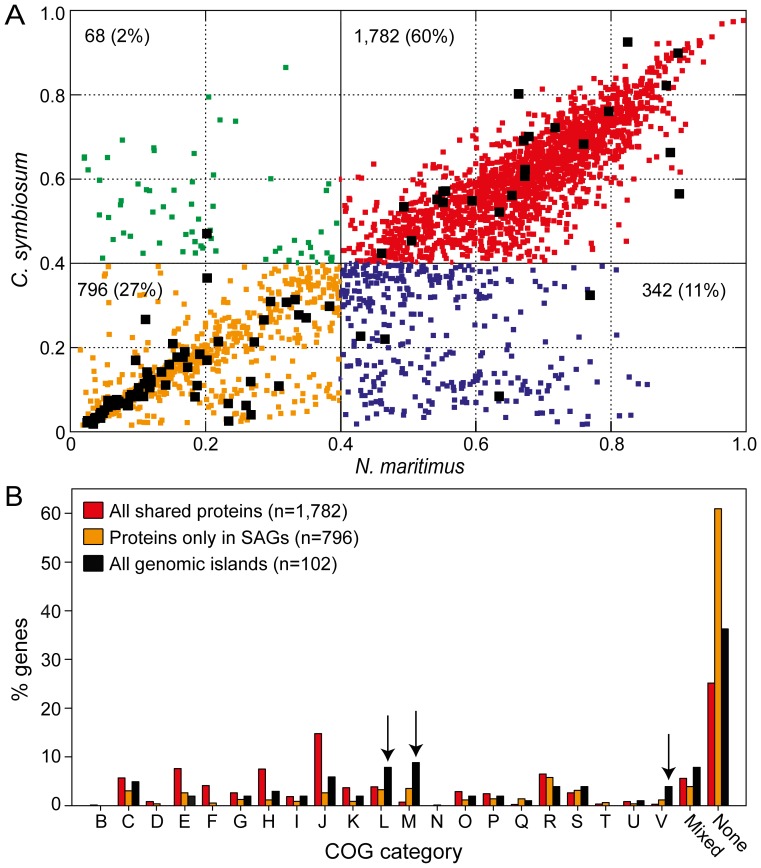
Homology and characterization of proteins from MGI single amplified genomes (SAGs) and thaumarchaea marine cultures. BLAST Score Ratio (BSR) analysis of the non-redundant protein set from 37 MGI SAGs (n = 2,988) (**A**), and characterization of selected homolog protein groups using Clusters of Orthologous Groups (COG) categories (**B**). BSR scores >0.4 (∼30% protein identity) are considered homologous. Proteins are color coded by homology pattern: red, shared among all genomes; blue, shared among SAGs and *N. maritimus*; green, shared among SAGs and *C. symbiosum*; yellow, not homologous to either culture. Proteins identified as a genomic island are represented by black squares. Arrows indicate enriched genomic island COG categories. COG categories: B, chromatin structure and dynamics; C, energy production and conversion; D, cell cycle control, mitosis, and meiosis; E, amino acid transport and metabolism; F, nucleotide transport and metabolism; G, carbohydrate transport and metabolism; H, coenzyme transport and metabolism; I, lipid transport and metabolism; J, translation; K, transcription; L, replication, recombination, and repair; M, cell wall/membrane biogenesis; N, cell motility; O, posttranslational modification, protein turnover, and chaperones; P, inorganic ion transport and metabolism; Q, secondary metabolite biosynthesis, transport, and catabolism; R, general function prediction only; S, function unknown; T, signal transduction mechanisms; U, intracellular trafficking and secretion; V, defense mechanisms; Mixed (multiple categories); None (no COG category). *C. symbiosum*, *Cenarchaeum symbiosum*; *N. maritimus*, *Nitrosopumilus maritimus*.

A large proportion of protein sequences from MGI SAGs were found to share homology with *C. symbiosum* and *N. maritimus* (Figure 4A). MGI SAGs were also found to share more homologous protein sequences with *N. maritimus* than *C. symbiosum*, with the former representing a free-living form of thaumarchaea. However, 27% of MGI SAG protein sequences had BSR ratios less than 0.4 when compared to sequences from either of the two cultures, suggesting they are unique to dark ocean thaumarchaea (Figure 4A). The ‘no COG category assignment’ (i.e. none) was found to characterize the largest group of SAG-only proteins (Figure 4B). The majority of sequences were annotated as hypothetical or conserved hypothetical, thus making it difficult to interpret the role of these proteins within the dark ocean thaumarchaea. Proteins that recruited no 454 metagenome sequences from the sampling environment, often termed genomic islands (GIs), were identified within MGI SAGs (Figure 4). Interestingly, the set of GI proteins was enriched in genes associated with COG categories L (Replication, Recombination, Repair), M (Cell Wall/Membrane Biogenesis), and V (Defense Mechanisms) (Figure 4B). This pattern in GI characterization has been noted in other marine taxa as well, and has been attributed to increased hypervariability among proteins associated with cell surfaces and cell recognition systems, which may function to reduce viral attachment [52]–[54] or recognition by grazers [55], [56]. These hypervariable genes may also function to mask genetic discontinuities detected within environmental gene sequences used to separate microorganisms into ecotypes [57]. Thus, this pattern of hypervariability among proteins functioning to protect the cell from infection or predation may be a universal feature among marine microbes.

### Summary

Single cell whole genome analysis of dark ocean thaumarchaea provided further evidence for their metabolic capacity for aerobic ammonia oxidation and urea hydrolysis for energy and/or biosynthesis, and utilization of inorganic and possibly organic carbon sources. Although MGI SAGs shared several metabolic features identified in *C. symbiosum* and *N. maritimus*, we found distinct depth distributions of genomic SAG sequences, suggesting genetic divergence between epipelagic and dark ocean thaumarchaea. The sequenced SAGs contain numerous genes with low homology to either of the two available marine thaumarchaea cultures, thus significantly expanding the known genetic repertoire of marine thaumarchaea. Similar to prior reports on marine bacteria, we found that dark ocean thaumarchaea contain hypervariable regions, with the majority of identifiable genes on these regions likely involved in cellular defenses. Our findings provide important insights into the metabolic potential, genetic variability, and vertical distribution of the globally important thaumarchaea in the ocean.

## Supporting Information

Figure S1 Alignment of nitrite reductase (*nirK*) and multicopper oxidase (MCO) gene sequences. Sequences recovered from archaeal and bacterial isolates, environmental samples, and selected single amplified genomes (SAGs) were used in the alignment. South Atlantic SAGs: AAA001-A19, AAA003-I22, AAA007-M20, and AAA008-M23; North Pacific SAGs: AAA288-E09, AAA288-G06, AAA288-K09, and AAA2888-N08. Identical (dark grey) and similar (light grey) amino acid residues are indicated, as well as locations of copper coordinating residues (arrows), as previously reported by Bartossek *et al.* [41]. Boxes surrounding residues and labeled “F” (forward) and “R” (reverse) indicate regions used for primer design. Sequences from *Nitrosopumilus maritimus* are prefaced by “Nmar” and *Cenarchaeum symbiosum* are prefaced by “CENSYa”.
(TIF)

Figure S2 Phylogeny of ammonia monooxygenase (*amoA*) sequences from South Atlantic (red) and North Pacific (blue) archaeal single amplified genomes (SAGs). The tree was inferred using maximum likelihood in RAxML and bootstrap (1000 replicates) values ≥50% are indicated at nodes. The tree was rooted with *Candidatus* ‘Nitrosocaldus yellowstonii’ (EU239961). Filed circles next to SAG *amoA* sequences indicate successful amplification of *nirK* genes from the same SAG.
(TIF)

Figure S3 Phylogeny of nitrite reductase (*nirK*) gene sequences from South Atlantic (red) and North Pacific (blue) thaumarchaea single amplified genomes (SAGs). The tree was inferred using maximum likelihood in RAxML and bootstrap (1000 replicates) values ≥50% are indicated at nodes. The *nirK* tree was rooted with multicopper oxidase (MCO) gene sequences.
(TIF)

Table S1 (PDF)

Table S2 (PDF)

Table S3 (PDF)

Table S4 (PDF)

Table S5 (PDF)

Table S6 (PDF)

Table S7 (PDF)
