# Supplementary figures and images for: Genomic and Metabolic Diversity of Marine Group I Thaumarchaeota in the Mesopelagic of Two Subtropical Gyres
Source: PLoS One. 2014 Apr 17;9(4):e95380. doi: 10.1371/journal.pone.0095380 (PMC3990693; doi:10.1371/journal.pone.0095380)

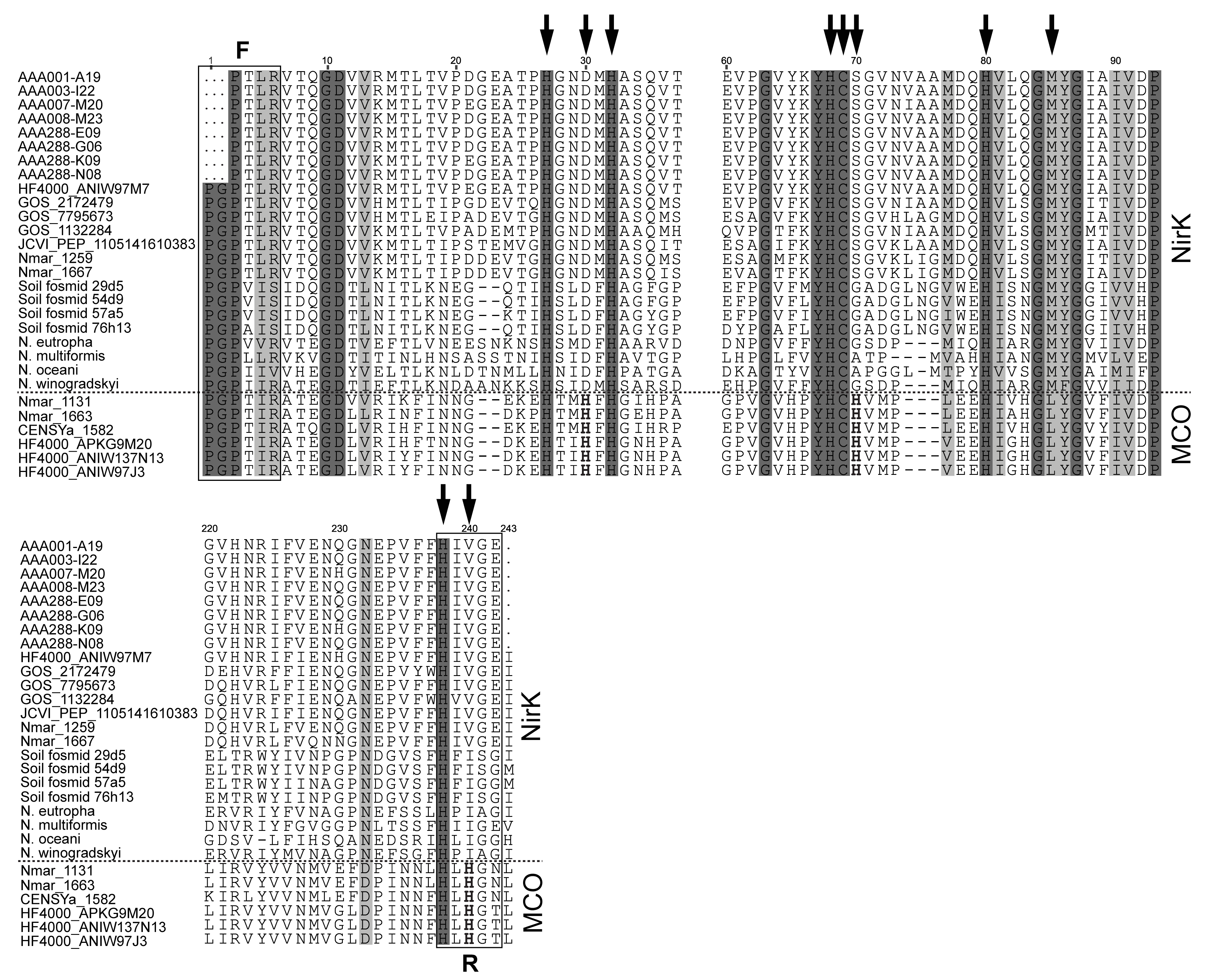

Supplement: Figure S1 — Alignment of nitrite reductase (nirK) and multicopper oxidase (MCO) gene sequences. Sequences recovered from archaeal and bacterial isolates, environmental samples, and selected single amplified genomes (SAGs) were used in the alignment. South Atlantic SAGs: AAA001-A19, AAA003-I22, AAA007-M20, and AAA008-M23; North Pacific SAGs: AAA288-E09, AAA288-G06, AAA288-K09, and AAA2888-N08. Identical (dark grey) and similar (light grey) amino acid residues are indicated, as well as locations of copper coordinating residues (arrows), as previously reported by Bartossek et al. [41]. Boxes surrounding residues and labeled “F” (forward) and “R” (reverse) indicate regions used for primer design. Sequences from Nitrosopumilus maritimus are prefaced by “Nmar” and Cenarchaeum symbiosum are prefaced by “CENSYa”. (TIF) [file pone.0095380.s001.tif]

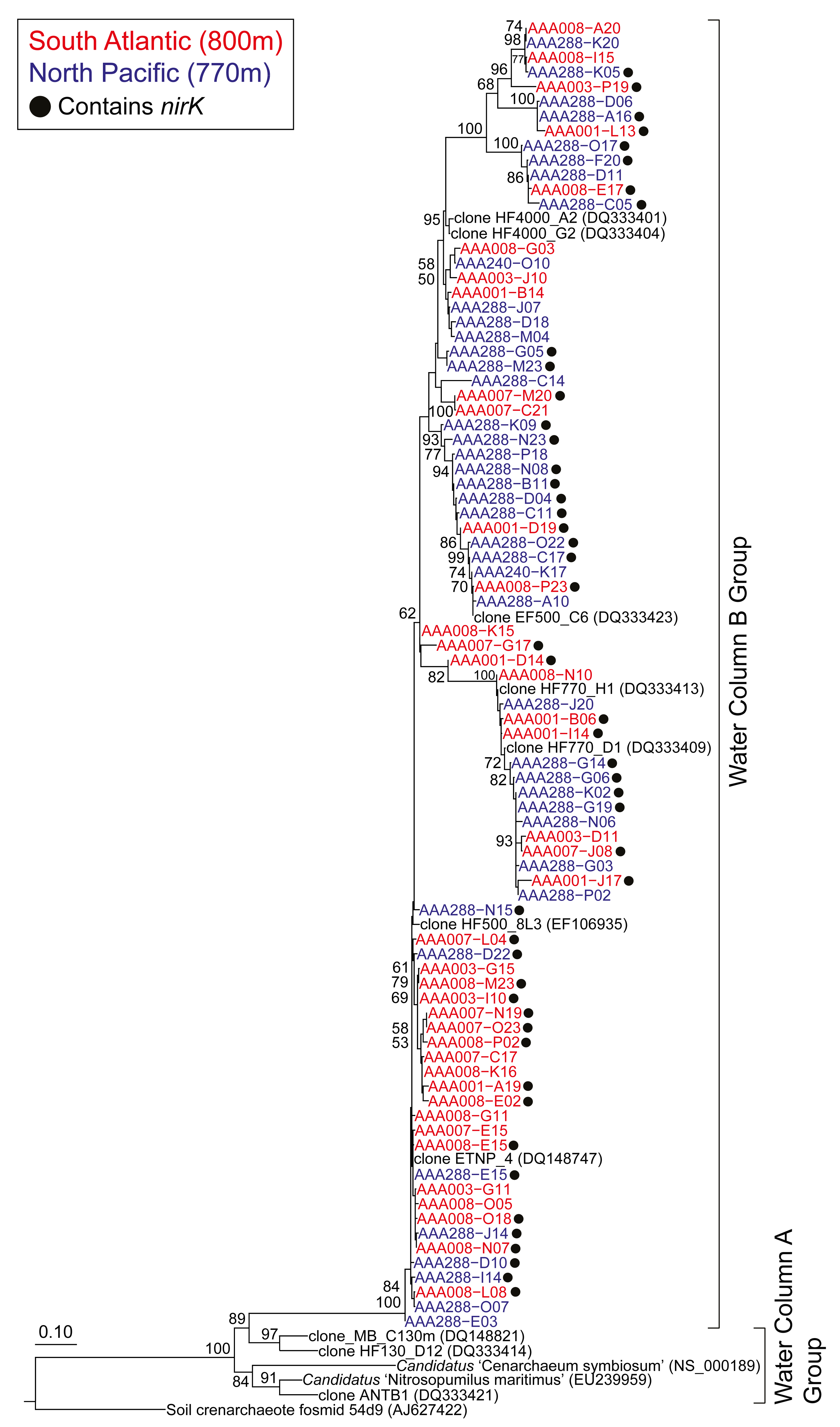

Supplement: Figure S2 — Phylogeny of ammonia monooxygenase (amoA) sequences from South Atlantic (red) and North Pacific (blue) archaeal single amplified genomes (SAGs). The tree was inferred using maximum likelihood in RAxML and bootstrap (1000 replicates) values ≥50% are indicated at nodes. The tree was rooted with Candidatus ‘Nitrosocaldus yellowstonii’ (EU239961). Filed circles next to SAG amoA sequences indicate successful amplification of nirK genes from the same SAG. (TIF) [file pone.0095380.s002.tif]

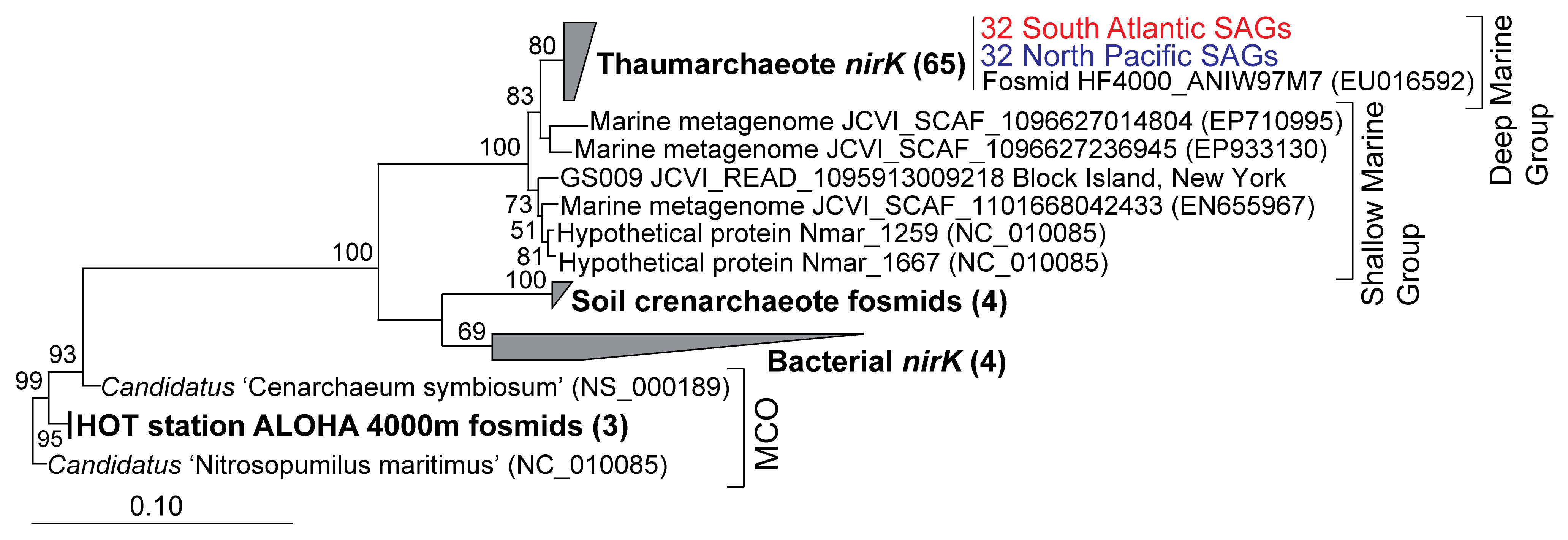

Supplement: Figure S3 — Phylogeny of nitrite reductase (nirK) gene sequences from South Atlantic (red) and North Pacific (blue) thaumarchaea single amplified genomes (SAGs). The tree was inferred using maximum likelihood in RAxML and bootstrap (1000 replicates) values ≥50% are indicated at nodes. The nirK tree was rooted with multicopper oxidase (MCO) gene sequences. (TIF) [file pone.0095380.s003.tif]
