# Supplementary material for: Genomic and Metabolic Diversity of Marine Group I Thaumarchaeota in the Mesopelagic of Two Subtropical Gyres
Source: PLoS One. 2014 Apr 17;9(4):e95380. doi: 10.1371/journal.pone.0095380 (PMC3990693; doi:10.1371/journal.pone.0095380)
Supplement: Table S1 — (PDF) [file pone.0095380.s004.pdf]

Table S1. PCR primers and conditions for screening of archaeal single amplified genomes (SAGs).

Primer	Sequence (5′ → 3′)	Annealing temp (°C)	Gene	Metabolic process	Reference
**SSU rRNA PCR primers**					
parch_519F	CAGCMGCCGCGGTAA	50	SSU rRNA	NA	Ovreas *et al.* , 1997
Arch_915R	GTGCTCCCCCGCCAATTCCT				DeLong, 1992
Arch_345F	CCTAYGGGGYGCASCAG	57	SSU rRNA	NA	Gantner *et al.* , 2010
Arch_1000R	GGCCATGCACYWCYTCTC				
**Metabolic gene PCR primers**					
Arch-amoAF	STAATGGTCTGGCTTAGACG	50	*amoA*	Ammonia oxidation	Francis *et al.* , 2005
Arch-amoAR	GCGGCCATCCATCTGTATGT				
NirKMF	CCWGGWCCAACHYTDAGA	50	*nirK*	Nitrite reduction	This study
NirkMR	ATTTCWCCDACAAYRTG				
nifHF	GGHAARGGHGGHATHGGNAARTC	55	*nifH*	Nitrogen fixation	Mehta *et al.* , 2003
nifHR	GGCATNGCRAANCCVCCRCANAC				

References:

Ovreas L, Forney L, Daae F, Torsvik V (1997) Distribution of bacterioplankton in meromictic Lake Saelenvannet, as determined by denaturing gradient gel electrophoresis of PCR-amplified gene fragments coding for 16S rRNA. Appl Environ Microbiol 63: 3367-3373.

DeLong EF (1992) Archaea in coastal marine environments. Proc Natl Acad Sci USA 89: 5685-5689.

Gantner S, Andersson AF, Alonso-Sáez L, Bertilsson S (2010) Novel primers for 16S rRNA-based archaeal community analyses in environmental samples. J Microbiol Methods 84: 12-18.

Francis CA, Roberts KJ, Beman JM, Santoro AE, Oakley BB (2005) Ubiquity and diversity of ammonia-oxidizing archaea in water columns and sediments of the ocean. Proc Natl Acad Sci USA 102: 14683-14688.

Mehta MP, Butterfield DA, Baross JA (2003) Phylogenetic diversity of nitrogenase (nifH) genes in deep-sea and hydrothermal vent environments of the Juan de Fuca Ridge. Appl Environ Microbiol 69: 960-970.
