# Supplementary material for: Genomic and Metabolic Diversity of Marine Group I Thaumarchaeota in the Mesopelagic of Two Subtropical Gyres
Source: PLoS One. 2014 Apr 17;9(4):e95380. doi: 10.1371/journal.pone.0095380 (PMC3990693; doi:10.1371/journal.pone.0095380)
Supplement: Table S2 — (PDF) [file pone.0095380.s005.pdf]

Table S2. Single amplified genomes (SAGs) comprising Marine Group I (MGI) Thaumarchaeota SSU rRNA phylotypes. Phylotypes are defined as sequences having ≥ 99% similarity.

Phylotype	Station[1]	SAG sequence[2]	Accession no.
**Phylotype-1**	SA	**AAA007-J08**	HQ675763
**Phylotype-2**	SA	AAA001-B06	HQ675728
		AAA001-C02	HQ675732
		AAA001-D14	HQ675733
		AAA001-I14	HQ675738
		AAA001-J17	HQ675740
		AAA003-E14	HQ675744
		AAA008-E23	HQ675781
		AAA003-G07	HQ675746
		**AAA008-J11**	HQ675785
		AAA008-M21	HQ675791
		AAA008-N10	HQ675794
	HOT	AAA288-B04	HQ675806
		AAA288-E04	HQ675823
		AAA288-G03	HQ675830
		AAA288-G06	HQ675832
		AAA288-G14	HQ675833
		AAA288-G19	HQ675834
		AAA288-J20	HQ675840
		**AAA288-K02**	HQ675841
**Phylotype-3**	SA	AAA003-D11	HQ675743
		AAA003-P19	HQ675756
		AAA007-C21	HQ675759
		AAA007-M20	HQ675767
	HOT	**AAA288-A08**	HQ675803
		AAA288-D03	HQ675814
		AAA288-N06	HQ675847
		AAA288-P02	HQ675854
**Phylotype-4**	SA	AAA001-L13	HQ675742
		AAA007-G17	HQ675762
		**AAA008-A20**	HQ675774
		AAA008-I15	HQ675784
	HOT	AAA288-A16	HQ675805
		AAA288-D06	HQ675816
		AAA288-K05	HQ675842
		AAA288-K20	HQ675844
**Phylotype-5**	SA	AAA001-D19	HQ675734
		AAA003-J13	HQ675753
		AAA003-O17	HQ675755

		AAA008-K15	HQ675788
		AAA008-N14	HQ675795
		**AAA008-P23**	HQ675800
	HOT	AAA240-K17	HQ675801
		AAA288-A10	HQ675804
		AAA288-B11	HQ675808
		AAA288-C11	HQ675810
		**AAA288-C17**	HQ675812
		AAA288-D04	HQ675815
		AAA288-I17	HQ675836
		AAA288-N08	HQ675848
		AAA288-O22	HQ675853
		AAA288-P18	HQ675855
**Phylotype-6**	SA	**AAA008-L08**	HQ675790
**Phylotype-7**	SA	AAA001-A19	HQ675727
		AAA001-B14	HQ675729
		AAA001-B20	HQ675730
		AAA001-E03	HQ675735
		AAA001-E10	HQ675736
		AAA001-G21	HQ675737
		AAA001-I18	HQ675739
		AAA001-J20	HQ675741
		AAA003-E23	HQ675745
		AAA003-G11	HQ675747
		AAA003-G15	HQ675748
		AAA003-I10	HQ675750
		AAA003-I22	HQ675751
		AAA003-J10	HQ675752
		AAA003-O08	HQ675754
		AAA007-C17	HQ675758
		AAA007-E15	HQ675760
		AAA007-E18	HQ675761
		AAA007-L04	HQ675765
		AAA007-N15	HQ675768
		AAA007-N19	HQ675769
		AAA007-N23	HQ675770
		AAA007-O23	HQ675772
		AAA008-A08	HQ675773
		AAA008-B15	HQ675775
		AAA008-C15	HQ675776
		AAA008-E02	HQ675777
		AAA008-E15	HQ675859
		AAA008-E18	HQ675780

		AAA008-G03	HQ675782
		AAA008-K07	HQ675786
		AAA008-K16	HQ675789
		AAA008-M23	HQ675792
		**AAA008-N07**	HQ675793
		AAA008-O05	HQ675796
		AAA008-O18	HQ675797
		AAA008-P02	HQ675798
		AAA008-P03	HQ675799
	HOT	AAA240-O10	HQ675802
		AAA288-B07	HQ675807
		AAA288-C14	HQ675811
		AAA288-D10	HQ675817
		AAA288-D18	HQ675820
		AAA288-D22	HQ675821
		AAA288-E03	HQ675822
		AAA288-E09	HQ675824
		AAA288-E15	HQ675825
		**AAA288-F11**	HQ675827
		AAA288-G02	HQ675829
		AAA288-G05	HQ675831
		AAA288-I14	HQ675835
		AAA288-J07	HQ675837
		AAA288-J11	HQ675838
		AAA288-J14	HQ675839
		AAA288-K09	HQ675843
		AAA288-M04	HQ675845
		AAA288-M23	HQ675846
		AAA288-N15	HQ675849
		AAA288-N23	HQ675850
		AAA288-O07	HQ675851
**Phylotype-8**	SA	AAA008-E17	HQ675779
	HOT	**AAA288-C05**	HQ675809
		AAA288-D11	HQ675818
		AAA288-F20	HQ675828
		AAA288-O17	HQ675852
**Phylotype-9**	SA	**AAA008-G11**	HQ675783

[1]HOT, North Pacific station ALOHA; SA, South Atlantic.
[2]Sequences in bold were chosen as the representative sequence(s) in the SSU rRNA tree.
