# Supplementary material for: Genomic and Metabolic Diversity of Marine Group I Thaumarchaeota in the Mesopelagic of Two Subtropical Gyres
Source: PLoS One. 2014 Apr 17;9(4):e95380. doi: 10.1371/journal.pone.0095380 (PMC3990693; doi:10.1371/journal.pone.0095380)
Supplement: Table S3 — (PDF) [file pone.0095380.s006.pdf]

Table S3. Sequencing and assembly characteristics of Marine Group I (MGI) Thaumarchaeota single amplified genomes (SAGs).

SAG[1]	IMG Taxon ID	Station	Assembly size (Mbp)	No. of contigs	% Genome recovery[2]	Estimated genome size (Mbp)	No. protein coding genes[3]	%GC
**AAA001-A19**	2513237067	South Atlantic	0.76	223	33.0	2.30	891	35.7
**AAA007-N19**	2513237068		0.89	194	73.4	1.21	1098	35.6
**AAA007-O23**	2527291500		1.10	32	94.7	1.17	1354	35.7
AAA007-C21	2524023106		0.15	24	20.2	0.72	265	34.9
AAA007-E02	2524023111		0.31	43	31.9	0.96	559	35.3
AAA007-E15	2524023107		0.26	29	23.4	1.12	468	35.1
AAA007-G17	2524023108		0.20	31	7.5	2.73	373	35.9
AAA007-M20	2524023109		0.11	27	7.5	1.44	231	35.4
AAA007-N23	2524023110		0.08	17	3.2	2.54	155	35.1
AAA008-E15	2524023112		0.28	33	26.6	1.05	485	35.1
AAA008-E17	2524023113		0.22	25	9.6	2.27	374	35.4
AAA008-G03	2524023114		0.36	44	36.2	1.01	660	35.2
AAA008-M21	2524023115		0.20	18	13.8	1.44	353	33.6
AAA008-M23	2524023116		0.23	20	12.8	1.77	370	35.2
AAA008-N07	2524023117		0.16	20	9.6	1.69	293	36.5
AAA008-O05	2524023118		0.24	24	28.7	0.84	423	35.6
AAA008-O18	2524023119		0.21	36	22.3	0.93	417	34.1
AAA008-P02	2524023120		0.30	45	25.5	1.18	574	35.7
AAA008-P23	2524023121		0.33	48	24.5	1.35	615	34.7
**AAA288-I14**	2513237066	North Pacific	1.06	150	78.7	1.35	1404	35.3
**AAA288-J14**	2513237065		0.68	253	55.3	1.23	796	35.8
AAA288-C17	2524023122		0.39	51	27.7	1.39	681	34.9
AAA288-D03	2524023123		0.26	42	11.7	2.26	518	34.6
AAA288-D22	2524023124		0.39	46	42.6	0.92	704	35.2
AAA288-E09	2524023125		0.31	46	27.7	1.13	547	35.3
AAA288-G05	2524023126		0.55	49	37.2	1.47	926	35.7
AAA288-K02	2524023127		0.43	70	31.9	1.36	761	35.6
AAA288-K05	2524023128		0.29	35	27.7	1.04	487	35.4
AAA288-K20	2524023097		0.37	54	24.5	1.52	685	35.5
AAA288-M04	2524023098		0.32	49	25.5	1.25	608	35.6
AAA288-M23	2524023099		0.28	48	20.2	1.39	529	34.5
AAA288-N15	2524023100		0.14	27	17.0	0.85	300	35.3
AAA288-N23	2524023101		0.31	39	27.7	1.12	539	34.3
AAA288-O17	2524023102		0.03	9	2.1	1.55	80	34.5
AAA288-O22	2524023103		0.25	38	17.0	1.48	466	34.33
AAA288-P02	2524023104		0.65	58	34.0	1.91	1069	35.06
AAA288-P18	2524023105		0.43	73	39.4	1.10	793	35.68

[1]SAGs in bold are genomes with larger assembly sizes; all other SAGs were derived from Nextera-prepared sequence libraries and had smaller genome assembly sizes.

[2]Genome recovery estimates based on 94 conserved single copy genes (CSCG) present within 98% of 155 archaeal genomes from IMG.

[3]Number of protein coding sequences determined after removing contigs <2 kbp.
