# Supplementary material for: Genomic and Metabolic Diversity of Marine Group I Thaumarchaeota in the Mesopelagic of Two Subtropical Gyres
Source: PLoS One. 2014 Apr 17;9(4):e95380. doi: 10.1371/journal.pone.0095380 (PMC3990693; doi:10.1371/journal.pone.0095380)
Supplement: Table S4 — (PDF) [file pone.0095380.s007.pdf]

Table S4. Metagenomes used in the fragment recruitment analysis of Marine Group I (MGI) Thaumarchaeota single amplified genomes (SAGs), and marine isolates *Cenarchaeum symbiosum* and *Nitrosopumilus maritimus*.

File Name[1]	Location	Date	Latitude	Longitude	Depth (m)	No. of seqs[2]	Library Type
HF10_Small	HOT (station ALOHA)	10/07/02	22.750000	-158.000000	10	7,829	Sanger
HF70_Small		10/07/02	22.750000	-158.000000	79	10,979	Sanger
HF130_Small		10/06/02	22.750000	-158.000000	130	6,754	Sanger
HF200_Small		10/06/02	22.750000	-158.000000	200	8,241	Sanger
HF500_Small		10/06/02	22.750000	-158.000000	500	9,004	Sanger
HF770_Small		12/21/03	22.750000	-158.000000	770	11,474	Sanger
HF4000_Small		12/21/03	22.750000	-158.000000	4,000	11,213	Sanger
HF4000_Large		12/21/03	22.750000	-158.000000	4,000	13,395	Sanger
HOT186_25m		10/19/06	22.750000	-158.000000	25	516,241	454 GS FLX
HOT186_75m		10/19/06	22.750000	-158.000000	75	558,827	454 GS FLX
HOT186_110m		10/19/06	22.750000	-158.000000	110	407,101	454 GS FLX
HOT186_500m		10/19/06	22.750000	-158.000000	500	835,910	454 GS FLX
HOT215_25m		09/09/09	22.750000	-158.000000	25	943,226	454 Ti FLX
HOT215_500m		09/09/09	22.750000	-158.000000	500	1,142,919	454 Ti FLX
HOT215_770m		09/09/09	22.750000	-158.000000	770	1,173,678	454 Ti FLX
HOT215_1000m		09/09/09	22.750000	-158.000000	1,000	1,225,938	454 Ti FLX
j4_10	NESAP LineP	06/08/09	48.650000	-126.666667	10	13,302	Sanger
j4_500			48.650000	-126.666667	500	10,587	Sanger
j4_1000			48.650000	-126.666667	1,000	12,308	Sanger
j4_1300			48.650000	-126.666667	1,300	9,175	Sanger
j12_10		06/09/09	48.970000	-130.666667	10	11,473	Sanger
j12_500			48.970000	-130.666667	500	10,781	Sanger
j12_1000			48.970000	-130.666667	1,000	13,080	Sanger
j12_2000			48.970000	-130.666667	2,000	6,330	Sanger
j26_10		06/24/09	50.000000	-145.000000	10	10,065	Sanger
j26_500			50.000000	-145.000000	500	18,030	Sanger
j26_1000			50.000000	-145.000000	1,000	11,669	Sanger
j26_2000			50.000000	-145.000000	2,000	12,333	Sanger
a4_10		08/21/09	48.650000	-126.666667	10	12,055	Sanger
a4_500			48.650000	-126.666667	500	15,302	Sanger
a4_1000			48.650000	-126.666667	1,000	13,776	Sanger
a4_1300			48.650000	-126.666667	1,300	9,649	Sanger
a12_10		08/23/09	48.970000	-130.666667	10	11,107	Sanger
a12_500			48.970000	-130.666667	500	9,924	Sanger
a12_1000			48.970000	-130.666667	1,000	11,929	Sanger
a12_2000			48.970000	-130.666667	2,000	12,916	Sanger
a26_10		08/27/09	50.000000	-145.000000	10	10,125	Sanger
a26_500			50.000000	-145.000000	500	8,917	Sanger
a26_1000			50.000000	-145.000000	1,000	7,267	Sanger
f40010		02/04/10	48.650000	-126.666667	10	13,996	Sanger
f40500			48.650000	-126.666667	500	14,295	Sanger
f41000			48.650000	-126.666667	1,000	14,573	Sanger
f41300			48.650000	-126.666667	1,300	14,513	Sanger
f12010		02/06/10	48.970000	-130.666667	10	14,237	Sanger
f12200			48.970000	-130.666667	200	14,633	Sanger
f12500			48.970000	-130.666667	500	14,656	Sanger
GB	Guaymas Basin, Plume 1	07/18/04	27.018167	-111.417667	1,996	497,180	454 Ti FLX
GB	Guaymas Basin, Plume 2	07/11/04	27.016667	-111.415500	1,775	463,130	454 Ti FLX
SRR304684	ETSP (OMZ; Stn. #3)	06/16/08	-20.116667	-70.383333	15	451,356	454 GS FLX
SRR304656		06/16/08	-20.116667	-70.383333	65	337,971	454 GS FLX
SRR304668		06/16/08	-20.116667	-70.383333	500	455,219	454 GS FLX
SRR304683		06/16/08	-20.116667	-70.383333	800	137,427	454 GS FLX
SRR064444		06/16/08	-20.116667	-70.383333	50	307,278	454 GS FLX
SRR064446		06/16/08	-20.116667	-70.383333	85	518,412	454 GS FLX
SRR064448		06/16/08	-20.116667	-70.383333	110	328,380	454 GS FLX
SRR064450		06/16/08	-20.116667	-70.383333	200	434,546	454 GS FLX
SA	South Atlantic Gyre	11/27/07	-12.494833	-4.998667	800	404,457	454 Ti FLX
KM3	Ionian Sea, Station KM3	11/17/04	36.510556	15.676944	3,000	9,091	Sanger

[1]Metagenomes from the same depth were combined for plotting of abundances in Figure 2.

[2]Number of sequences used after quality trimming and processing.
