# Supplementary material for: Genomic and Metabolic Diversity of Marine Group I Thaumarchaeota in the Mesopelagic of Two Subtropical Gyres
Source: PLoS One. 2014 Apr 17;9(4):e95380. doi: 10.1371/journal.pone.0095380 (PMC3990693; doi:10.1371/journal.pone.0095380)
Supplement: Table S5 — (PDF) [file pone.0095380.s008.pdf]

**Table S5.** Genes for aerobic oxidation of ammonia identified in Marine Group I (MGI) Thaumarchaeota single amplified genomes (SAGs).

| **Enzyme** | AAA001-A19 | AAA007-N19 | AAA007-O23 | AAA288-I14 | AAA288-J14 |
|---|---|---|---|---|---|
| Ammonia monooxygenase, subunit A (1.14.99.39) | | ● | ● | ● | ● |
| Ammonia monooxygenase, subunit B | | ● | ● | ● | ● |
| Ammonia monooxygenase, subunit C | ● | ● | ● | | ● |
| Ubiquinol-cytochrome c reductase (1.10.2.2) | ● | ● | ● | | ● |
| Ammonia permease | ● | ● | | | |
| Copper-containing nitrite reductase (1.7.2.1) | ● | ● | ● | ● | ● |
| Copper cytochrome c oxidase (1.9.3.1) | | ● | ● | ● | ● |
| NADH-quinone oxidoreductase | ● | | ● | | ● |
| Copper binding protein, plastocyanin/azurin family | 1 | 3 | 4 | 3 | 1 |
| Multicopper oxidase | 2 | 1 | 2 | 2 | 1 |
