# Supplementary material for: Genomic and Metabolic Diversity of Marine Group I Thaumarchaeota in the Mesopelagic of Two Subtropical Gyres
Source: PLoS One. 2014 Apr 17;9(4):e95380. doi: 10.1371/journal.pone.0095380 (PMC3990693; doi:10.1371/journal.pone.0095380)
Supplement: Table S6 — (PDF) [file pone.0095380.s009.pdf]

Table S6. Central metabolism genes identified in Marine Group I (MGI) Thaumarchaeota single amplified genomes (SAGs). Genes in red were also not identified in *Nitrosopumilus maritimus* .

Enzyme/Pathway	AAA001-A19	AAA007-N19	AAA007-O23	AAA288-I14	AAA288-J14
**3-Hydroxyproprionate/4-Hydroxybutyrate Cycle**					
Acetyl-CoA carboxylase (6.4.1.2/6.3.4.14)	●		●		●
Malonyl-CoA reductase (1.2.1.75)					
Malonate semialdehyde reductase (1.1.1.298)					
3-Hydroxypropionyl-CoA synthetase (6.2.1.36)					
3-Hydroxypropionyl-CoA dehydratase (4.2.1.116)					
Acryloyl-CoA reductase (1.3.1.84)					
Propionyl-CoA carboxylase (6.4.1.3)	●		●		●
Methylmalonyl-CoA epimerase (5.1.99.1)	●	●	●	●	
Methylmalonyl-CoA mutase (5.4.99.2)	●	●	●	●	
Succinyl-CoA reductase (1.2.1.76)					
Succinate semialdehyde reductase (1.1.1-)	●	●	●	●	
4-Hydroxybutyryl-CoA synthetase (6.2.1-)	●	●	●	●	●
4-Hydroxybutyryl-CoA dehydratase (42.1.120/5.3.3.3)	●	●	●	●	●
Crotonyl-CoA hydratase (4.2.1-)		●	●	●	●
3-Hydroxybutyryl-CoA dehydratase (42.1.55/1.1.1.35)	●	●	●	●	●
Acetoacetyl-CoA ß-ketothiolase (2.3.1.9)	●	●	●	●	●
**Citric Acid Cycle (TCA)**					
citrate synthase (2.3.3.1)		●	●	●	
citrate lyase (beta subunit)/citryl-CoA lyase (4.1.3.6)	●		●	●	
aconitate hydratase (4.2.1.3)			●	●	
isocitrate dehydrogenase (1.1.1.42)		●	●		
oxoglutarate ferredoxin oxidoreductase (1.2.7.3)		●	●	●	
succinyl CoA-synthetase (6.2.1.5)	●	●	●	●	
succinate dehydrogenase (1.3.99.1)		●	●	●	●
fumurate hydratase (4.2.1.2)	●	●	●		●
malate dehydrogenase (1.1.1.37)		●		●	●
**Glycolysis/Embden–Meyerhof–Parnas (EMP)**					
glucokinase (2.7.1.2)					
glucose-6-phosphate isomerase/aldose 1-epimerase (5.3.1.9 / 5.1.3.3)		●	●		
6-phosphofructokinase (2.7.1.11)					
fructose 1,6-bisphosphate aldolase (4.1.2.13)	●		●	●	●
triosephosphate isomerase (5.3.1.1)	●	●		●	
glyceraldehyde-3-phosphate dehydrogenase (1.2.1.12)				●	
phosphoglycerate kinase (2.7.2.3)		●			
phosphoglycerate mutase (5.4.2.1)		●		●	●
enolase (4.2.1.11)		●			●
pyruvate kinase (2.7.1.40)					
**Pentose Phosphate Pathway**					
glucose-6-phosphate dehydrogenase (1.1.1.49)					
6-phosphogluconolactonase (3.1.1.31)					
6-phosphogluconate dehydrogenase (1.1.1.44)					
ribulose-phosphate 3-epimerase (5.1.3.1)					
ribose-5-phosphate isomerase (5.3.1.6)	●	●	●	●	●
transketolase (2.2.1.1)	●	●	●	●	●
transaldolase (2.2.1.2)		●	●	●	●
