# Supplementary material for: Genomic and Metabolic Diversity of Marine Group I Thaumarchaeota in the Mesopelagic of Two Subtropical Gyres
Source: PLoS One. 2014 Apr 17;9(4):e95380. doi: 10.1371/journal.pone.0095380 (PMC3990693; doi:10.1371/journal.pone.0095380)
Supplement: Table S7 — (PDF) [file pone.0095380.s010.pdf]

Table S7. Transporter genes identified in Marine Group I (MGI) Thaumarchaeota single amplified genomes (SAGs).

**Transporter/Type**	AAA001-A19	AAA007-N19	AAA007-O23	AAA288-I14	AAA288-J14
**ABC Transporters**					
Mineral and Organic Ion					
Iron (III) transport system substrate biding protein					●
Phosphate and Amino Acid					
Phosphonate transport system substrate-binding protein	●		●	●	
Phosphonate transport system permease protein	●	●	●		
Phosphonate transport system ATP-binding protein			●		
Oligosaccharide and Polyol Transporters					
Multiple sugar transport system permease protein					●
ABC-type 2 and other Transporters					
Antibiotic transport system permease protein	●		●	●	●
Antibiotic transport system ATP-binding protein	●		●	●	
Lipoprotein-releasing system permease protein		●		●	
Lipoprotein-releasing system ATP-binding protein		●		●	
Metal Cation, Iron Siderophore and Vitamin B12					
Zinc transport system permease protein	●		●	●	
Zinc transport system ATP-binding protein	●		●	●	
